# A New Method to Determine Antigen-Specific CD8^+^ T Cell Activity *in Vivo* by Hydrodynamic Injection

**DOI:** 10.3390/biom2010023

**Published:** 2012-01-05

**Authors:** Urvashi Rai, Jing Huang, Satish Mishra, Xiangming Li, Takayuki Shiratsuchi, Moriya Tsuji

**Affiliations:** 1 HIV and Malaria Vaccine Program, The Aaron Diamond AIDS Research Center, Affiliate of the Rockefeller University, New York, NY 10016, USA; Email: urai@adarc.org (U.R.); jhuang@adarc.org (J.H.); xli@adarc.org (X.L.); Takayuki.Shiratsuchi@otsuka-us.com (T.S.); 2 Michael Heidelberger Division, Department of Pathology, New York University School of Medicine, New York, NY 10016, USA; Email: Satish.Mishra@nyumc.org

**Keywords:** CD8^+^ T cells, Hydrodynamic tail vein, liver, malaria, plasmid DNA, luciferase, bioluminescence, recombinant adenovirus

## Abstract

Hydrodynamic tail vein (HTV) delivery is a simple and rapid tail vein injection method of a high volume of naked plasmid DNA resulting in high levels of foreign gene expression in organs, especially the liver. Compared to other organs, HTV delivery results in more than a 1000-fold higher transgene expression in liver. After being bitten by malaria-infected mosquitoes, malaria parasites transiently infect the host liver and form the liver stages. The liver stages are known to be the key target for CD8^+^ T cells that mediate protective anti-malaria immunity in an animal model. Therefore, in this study, we utilized the HTV delivery technique as a tool to determine the *in vivo* cytotoxic effect of malaria antigen-specific CD8^+^ T cells. Two weeks after mice were immunized with recombinant adenoviruses expressing malarial antigens, the immunized mice as well as naïve mice were challenged by HTV delivery of naked plasmid DNA co-encoding respective antigen together with luciferase using dual promoters. Three days after the HTV challenge, non-invasive whole-body bioluminescent imaging was performed. The images demonstrate *in vivo* activity of CD8^+^ T cells against malaria antigen-expressing cells in liver.

## 1. Introduction

In a complex plasmodial cycle, the liver stage, in which parasites reside in the liver, represents an important stage for the cell-mediated immunity to take place in the host. The role of CD8^+^ T cells in protective immunity against the liver stages has been well established in a rodent model. Evidence from *in vivo* depletion of CD8^+^ T cells in mice clearly demonstrated its protective role against the liver stages [1,2]. Furthermore, adoptive transfer studies corroborated the findings of protective CD8^+^ T cell functions [3,4]. Finally, we have previously shown that a single immunizing dose of a recombinant adenovirus (rAd) expressing a circumsporozoite (CS) antigen of *Plasmodium yoelii*, AdPyCS, induced a robust PyCS-specific CD8^+^ T cell response; moreover, a strong protective anti-malaria immunity, which is mediated by CD8^+^ T cells [5]. 

We have chosen two pre-erythrocytic antigens, as model antigens, to test the *in vivo* function of CD8^+^ T cells in the current study. The CS protein, which is a major surface protein of malarial sporozoite, has been well-characterized and shown to mediate protective immunity against malaria [3,4,5,6]. CS protein-based vaccine, called RTS,S is undergoing Phase III trial [7]. Cell traversal protein of *Plasmodium* ookinetes and sporozoites (CelTOS), another pre-erythrocytic antigen, is shown to be recognized by T cells of at least 50% of human volunteers immunized with irradiated sporozoites of *P. falciparum* [8]. This CelTOS is a microneme protein secreted by sporozoites and shown to mediate protective anti-malaria immunity in a mouse model [9].

After the gene expression in muscle was observed upon intramuscular injection of naked plasmid DNA [10], a non-viral delivery of nucleic acids by injecting a large volume of solutions was performed [11,12]. Then, a simple technique called Hydrodynamics-based gene transfection was developed in the late 90s [13,14]. Using this technique, a rapid tail vein injection of a high volume of naked plasmid DNA was performed, leading to high levels of foreign gene expression in organs, especially the liver. This delivery method, called Hydrodynamic Tail Vein (HTV) delivery, is simple and achieves 40% of liver transfection [14]. 

In our studies, we established a tool to measure the *in vivo* cytotoxic effect of malaria specific CD8^+^ T cells using a Hydrodynamic Tail Vein (HTV) injection. For this purpose, mice were first immunized with rAd expressing malaria antigens, described above, to mount malaria-specific CD8^+^ T cells. A group of immunized mice, as well as naïve mice, then received plasmid DNA encoding respective antigens together with luciferase gene through HTV delivery. Finally, all the mice challenged with the DNA by HTV delivery were subjected to non-invasive whole body bioluminescent imaging to determine the level of luciferase expression in their livers and assess the function of malaria-specific CD8^+^ T cells response *in vivo*. 

## 2. Experimental Section

### 2.1. Plasmid Vector

**Figure 1:**
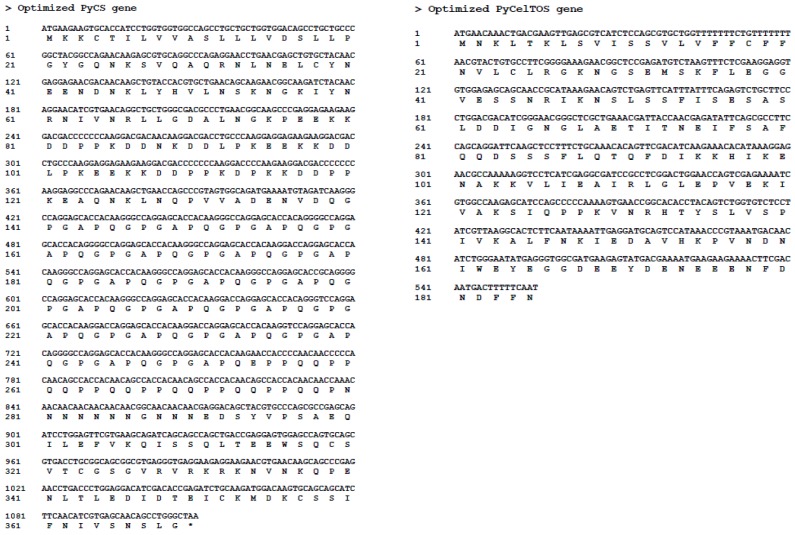
Codon optimized sequences of PyCS and PyCelTOS.

The genes coding for *P. yoelii* CS protein and *P. yoelii* CelTOS protein were first codon optimized, as shown in Figure 1, and then linked to the luciferase gene using a linker (sequence: PGILASQSTCRHASLRPIQ). The constructs were then amplified and cloned into a vector plasmid, pCMV-MCS (Agilent Technologies, Stratagene Products Division, La Jolla, CA, USA). The final constructs were verified by sequencing. The plasmid DNA, named DNAPyCS-Luc and DNAPyCelTOS-Luc, were purified with a Midi purification kit (Qiagen, Valencia, CA, USA). 

### 2.2. Recombinant Adenoviruses

Recombinant Adenoviruses (rAds) expressing *P. yoelii* CS protein, AdPyCS, and *P. yoelii* CelTOS protein, AdPyCelTOS, were generated previously [15]. Briefly, after both PyCS and PyCelTOS genes were codon optimized (Figure 1), the optimized fragments were cloned into a shuttle vector, pShuttle-CMV5, and the PmeI linearized shuttle vector was introduced into *E. coli* strain of BJ5183 that harbored the adenoviral backbone vector, pAdEasy-1 (Agilent Technologies, Stratagene Products Division, La Jolla, CA, USA). Recombinant Ad plasmids were transfected into AD-293 cells (Stratagene, Cedar Creek, TX, USA) to generate rAds. Finally, rAds were amplified and subsequently purified by CsCl gradient ultracentrifugation, previously described [16]. Virus particle (v.p.) was calculated based on O.D._260_ (1 OD_260_ = 1.25 × 10^12^ v.p./mL).

### 2.3. Animals

Eight-ten weeks old female BALB/c mice were obtained from Taconic Farms (Hudson, NY, USA). Animals were maintained in the Laboratory Animal Research Center of the Rockefeller University following standard provisions. The animal protocols, #8065 and #10095, were approved by the Institutional Animal Care and Use committee at the Rockefeller University. 

### 2.4. Immunizations

Group of three mice were immunized intramuscularly with 1 × 10^10^ virus particles (v.p.) of AdPyCS or AdPyCelTOS. Two weeks later, the mice received the plasmid DNA by HTV injection in less than 5 seconds after the plasmid DNA was diluted in PBS in a total volume of 2 mL. 

### 2.5. *In Vivo* Depletion of CD8+ T Cells

Group of three mice were administered with monoclonal antibodies against CD8^+^ T cells (YTS 169) (Harlan Bioproducts For Science Inc, Madison, WI, USA) to deplete CD8^+^ T cell from previously immunized mice. Briefly, 500 μg of YTS 169 diluted in PBS was injected intra-peritoneally at 3 and 1 day prior to the DNA challenge. We confirmed that this anti-CD8^+^ T cell antibody administration regimen resulted in the depletion of more than 95% of CD8^+^ T cells among splenocytes by FACS analysis (data not shown). 

### 2.6. Luciferase Expression by Noninvasive Bioluminescent Imaging

Three days after the HTV injection, the images of the luciferase expression in mouse liver was monitored using Caliper Life LifeSciences IVIS®Lumina/Living Image (Caliper LifeScience, Hopkinton, MA, USA). Briefly, after anesthetizing the mice, 200 μL of 15 mg/mL D-luciferin (Gold Biotechnology, St Louis, MO, USA) was injected intra-peritoneally, and the whole body *in vivo* imaging analysis was performed for 30 sec to 2 min, using *in vivo* imaging system (IVIS®Lumina). Luciferase expression data were then quantified using the Living Image software (Caliper LifeScience) in a fixed region of interest (ROI) in terms of photons/sec/cm^2^/sr. 

### 2.7. Statistical Analysis

Statistical analysis of experimental and control data was evaluated by Student’s *t*-test. A value of *P* < 0.01 was considered statistically significant.

## 3. Results

### 3.1. Level of Luciferase Expression *in vivo* after HTV Injection of Various Doses of Plasmid DNA Co-Encoding Malaria Antigen and Luciferase

**Figure 2:**
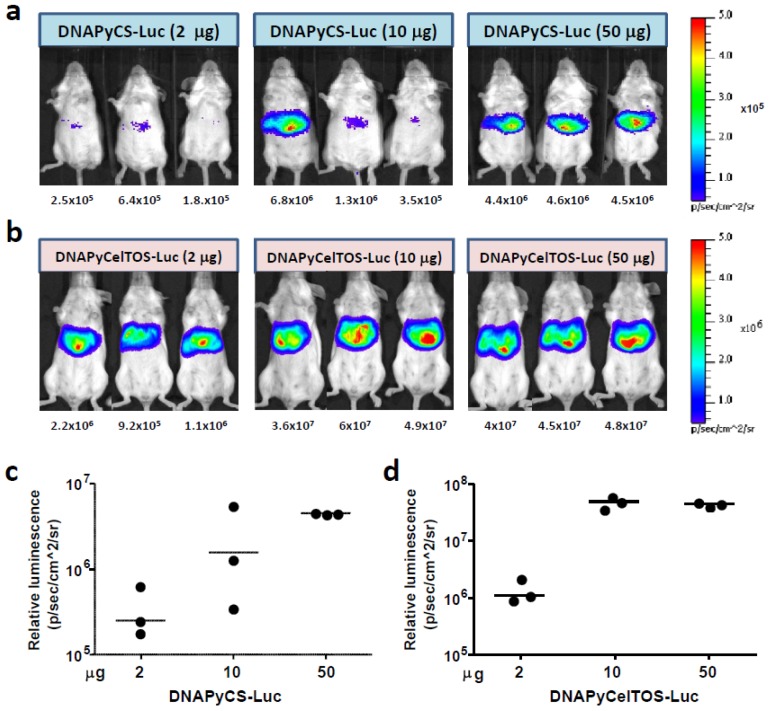
Luciferase expression in the liver after Hydrodynamic tail vein (HTV) injection of various doses of each plasmid DNA, co-encoding genes for a malaria antigen and luciferase. (**a**) and (**b**) Non-invasive bioluminescence imaging depicts the luciferase expression in the liver after HTV injection of 2, 10, and 50 μg of plasmid DNA co-encoding genes for PyCS antigen and luciferase in (**a**) and plasmid DNA encoding genes for PyCelTOS antigen and luciferase in (**b**). Three day after HTV injection, mice were anesthetized and injected with D-luciferin, and the luciferase intensity was optically imaged. The numbers below indicate the bioluminescent signal intensity in the region of interest (ROI), quantified as photons/sec/cm^2^/sr. (**c**) and (**d**) The graphs show the bioluminescent signal intensity in the ROI, as calculated by photons/sec/cm^2^/sr, of the same mice observed in (**a**) and (**b**), respectively.

In order to determine the level of the luciferase expression in the liver after HTV injection of different doses of plasmid DNA, we injected various doses, 2 μg, 10 μg and 50 μg, of plasmid DNA co-encoding PyCS and luciferase (DNAPyCS-Luc), or plasmid DNA co-encoding PyCelTOS and luciferase (DNAPyCelTOS-Luc). Three days post HTV injection, we performed a non-invasive whole body bioluminescent imaging using IVIS (Caliper LifeSciences) and found that the HTV injection of 50 μg of DNAPyCS-Luc (Figure 2a and 2c), as well as 10–50 μg of DNAPyCelTOS-Luc (Figure 2b and 2d) induced the highest level of luciferase expression in the liver. It is noteworthy that the luciferase expression after DNAPyCS-Luc challenge is lower than that after DNAPyCelTOS-Luc challenge. This difference may be due to two reasons. Firstly, the translation efficiency of the PyCS coding region may be decreased compare to that of PyCelTOS, due to the unstable nature (it contains a highly repetitive sequence in the middle) and the PyCS gene is twice the size of the PyCelTOS gene. Secondly, luciferase is fused with PyCS antigen with a short linker, therefore the structure of PyCS may possibly affect the level of luciferase expression. In fact, we observed a lower expression of PyCS protein compared to PyCelTOS protein upon *in vitro* transfection of the corresponding plasmids. Nevertheless, in the subsequent experiments, we decided to choose 30 μg and 3 μg of DNAPyCS-Luc and DNAPyCelTOS-Luc, respectively, for the HTV injection. 

### 3.2. Inhibition of DNAPyCS-Luc Induced Luciferase Expression by a Single Immunizing Dose of AdPyCS, but not AdPyCelTOS

The level of luciferase expression upon HTV injection of DNAPyCS-Luc was determined in mice immunized with AdPyCS or AdPyCelTOS, compared to those in naïve mice. For this purpose, we immunized a group of mice with AdPyCS or AdPyCelTOS, and 2 weeks later, we challenged the immunized mice, as well as naïve mice, with HTV injection of DNAPyCS-Luc. 

**Figure 3:**
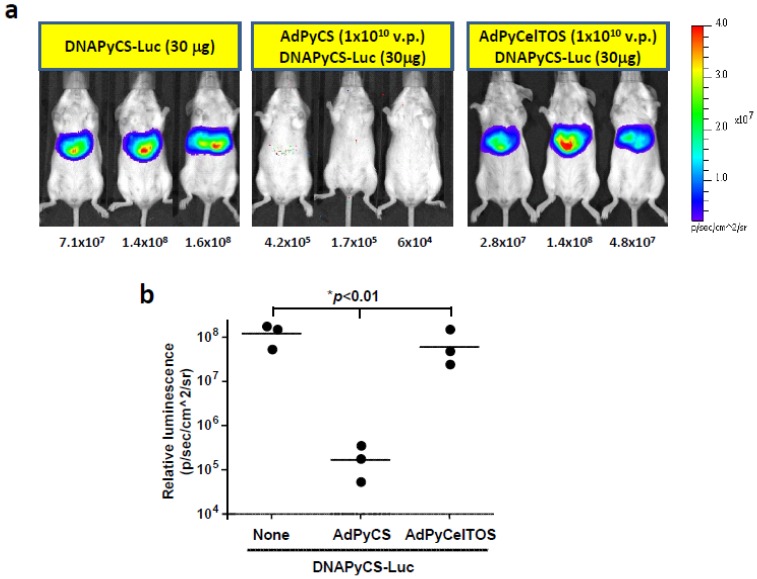
Luciferase expression in AdPyCS-immunized mice, AdPyCelTOS-immunized mice, or naïve mice, upon HTV injection of DNAPyCS-Luc. (**a**) Noninvasive bioluminescence image shows the inhibition of DNAPyCS-Luc induced luciferase expression in mice by a prior single immunizing dose of AdPyCS, but not of AdPyCelTOS. Three day after the HTV injection with DNAPyCS-Luc, mice were anesthetized and injected with D-luciferin, and the luciferase intensity was optically imaged; (**b**) Quantification of the bioluminescent signal intensity in AdPyCS-immunized mice, AdPyCelTOS-immunized mice, or naïve mice, upon HTV injection of DNAPyCS-Luc.

Non-invasive bioluminescent images have shown the complete inhibition of luciferase expression in the liver of a group of mice receiving a single immunizing dose of AdPyCS, but not AdCelTOS (Figure 3a and 3b). This indicates that the level of luciferase expression induced by DNAPyCS-Luc was inhibited by an antigen-specific fashion. 

### 3.3. Inhibition of DNAPyCelTOS-Luc Induced Luciferase Expression by a Single Immunizing Dose of AdPyCelTOS, but not AdPyCS

**Figure 4:**
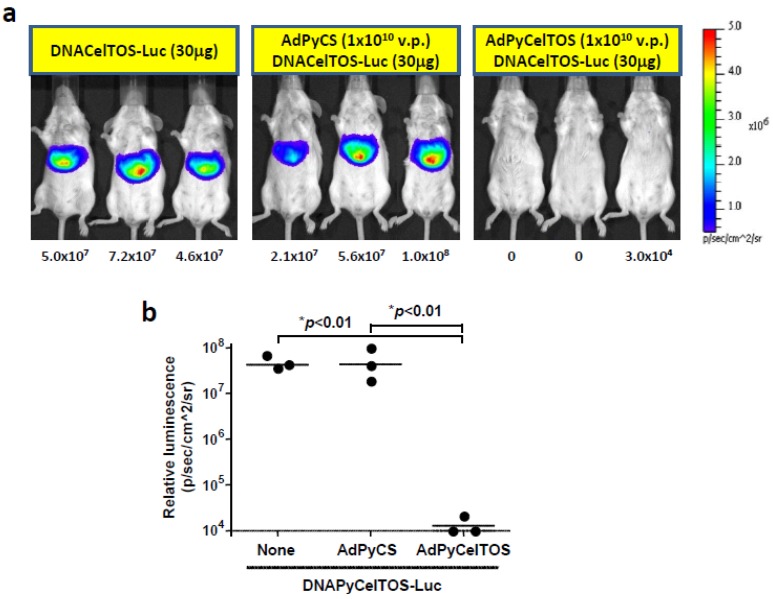
Luciferase expression in AdPyCS-immunized mice, AdPyCelTOS-immunized mice, or naïve mice, upon HTV injection of DNAPyCelTOS-Luc. (**a**) Noninvasive bioluminescence image shows the inhibition of DNAPyCelTOS-Luc induced luciferase expression in mice by a prior single immunizing dose of AdPyCelTOS, but not of AdPyCS. Three days after the HTV injection with DNAPyCelTOS-Luc, mice were anesthetized and injected with D-luciferin, and the luciferase intensity was optically imaged; (**b**) Quantificationof the bioluminescent signal intensity in AdPyCS-immunized mice, AdPyCelTOS-immunized mice, or naïve mice, upon HTV injection of DNAPyCelTOS-Luc.

In order to determine whether the antigen-specific inhibition of the level of luciferase expression is unique to the PyCS antigen, we also determined the luciferase expression upon HTV injection of DNAPyCelTOS-Luc in the second set of experiments. Briefly, we first immunized a group of mice with AdPyCS or AdPyCelTOS, and two weeks later, we challenged these mice, as well as naïve mice, with HTV injection of DNAPyCelTOS-Luc. In corroboration with our results from HTV injection of DNAPyCS-Luc (Figure 3), only a group of mice receiving a single immunizing dose of AdPyCelTOS, but not AdPyCS, could inhibit the level of luciferase expression induced by DNAPyCelTOS-Luc, as shown in Figure 4a and 4b. Thus, the inhibition of luciferase expression induced by plasmid DNA coencoding malaria antigen and luciferase is due to the immune response elicited by immunization of a rAd expressing the same antigen. 

### 3.4. CD8^+^ T Cell-Mediated Inhibition of Luciferase Expression Induced by HTV Injection of DNA Co-Encoding Malaria Antigen and Luciferase in Mice Immunized with a rAd Expressing the Antigen

**Figure 5:**
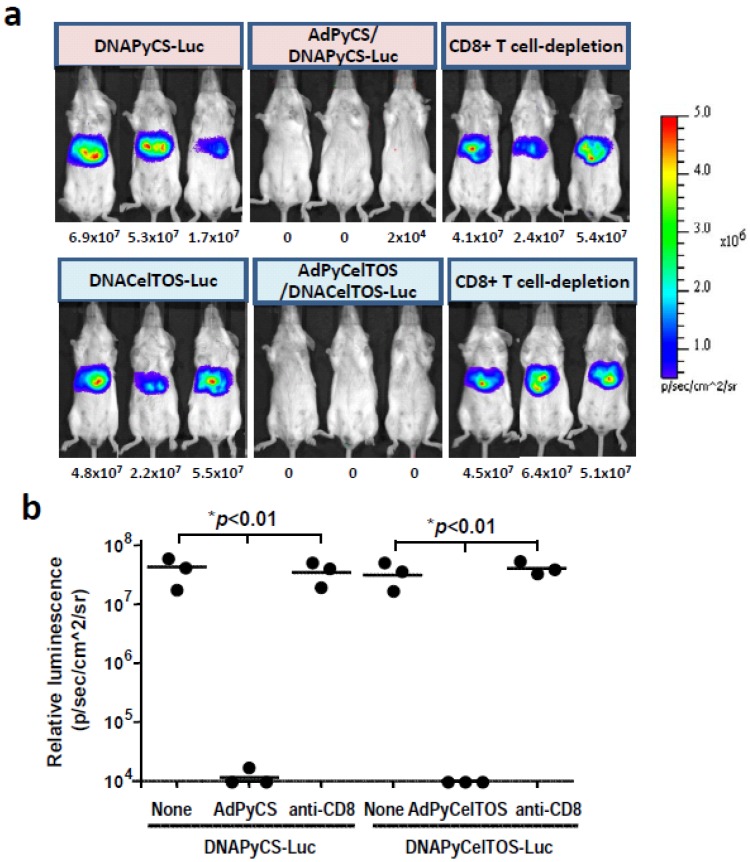
CD8^+^ T cell-mediated inhibition of luciferase expression in mice upon HTV injection of plasmid DNA, co-encoding genes for a malaria antigen and luciferase, by immunization with rAd expressing the antigen. (**a**) CD8^+^ T cell population was depleted from malaria vaccine-immunized mice before HTV injection with plasmid DNA co-encoding a malaria antigen and luciferase, and luciferase expression was assessed by noninvasive bioluminescence imaging; (**b**) The bioluminescent signal intensity was quantified in various groups of mice upon HTV injection of plasmid DNA co-encoding a malaria antigen and luciferase. The groups include naïve mice, malaria vaccine-immunized mice, and malaria vaccine-immunized mice that were depleted of CD8^+^ T cells *in vivo.*

In this study, we have shown that a single immunization of rAd expressing a malarial antigen could inhibit the level of luciferase expression in the liver of mice upon HTV injection with a plasmid DNA co-encoding the antigen and luciferase (Figure 3 and Figure 4). We have previously shown that a single immunizing dose of AdPyCS could elicit a robust CD8^+^ T cell response that can attack the liver stages of rodent malaria [5]. Therefore, we hypothesized that the inhibition of luciferase expression induced by the HTV injection with the DNA co-encoding the malaria antigen and luciferase could be due to malaria-specific CD8^+^ T cells elicited by the immunization with rAd expressing the same malaria antigen. To determine if this is the case, we depleted the CD8^+^ T cell subset from mice immunized with AdPyCS or AdPyCelTOS prior to challenge with DNAPyCS-Luc and DNAPyCelTOS-Luc by HTV injection, respectively. As shown in Figure 5a and 5b, the inhibition of luciferase expression in the liver of DNAPyCS-Luc-challenged mice observed after the AdPyCS immunization was completely abolished and the luciferase expression was recovered in mice depleted of CD8^+^ T cells. Similarly, the inhibition of luciferase expression induced by the HTV challenge with DNAPyCelTOS-Luc observed in mice immunized with AdPyCelTOS was abrogated by the CD8^+^ T cell depletion. These results clearly indicate that malaria antigen-specific CD8^+^ T cells induced by immunization with rAd expressing the antigen are responsible for inhibiting the luciferase expression in the liver of mice challenged with HTV injection of DNA co-encoding the antigen and luciferase. 

## 4. Discussion

A non-viral gene delivery platform has been shown to be the simple and efficient method of gene expression in various organs using rodent model [10,11,12,13,14,17,18]. With the novel concept of hydrodynamics-based delivery [13,14], the transgene expression has been observed in liver, lung, heart, kidney and also various species [17]. However, the highest gene expression has been achieved only in liver [13,14]. The hepatic transfer of DNA via HTV injection is a physical process, called hydroporation, in which membrane pores are generated by highly pressured solution in liver [17,18]. 

Hepatocytes are known to be non-professional antigen-presenting cells (APCs), and since hepatocytes do not express sufficient co-stimulatory molecules, they are not efficient at priming and inducing CD8^+^ T cells like professional APCs, including Kupffer cells and dendritic cells. However, when hepatocytes are infected with hepatotrophic virus, such as HCV, they can act as a target for the CD8^+^ T cells [19]. In fact, both human and murine CD8^+^ T cells are shown to recognize endogenously synthesized and processed virus proteins in association with MHC-class I molecules, and eliminate virus-infected hepatocytes [20,21]. Therefore, having taken advantage of the natures of hepatocytes that are capable of processing and presenting CD8^+^ epitopes and being recognized by epitope-specific CD8^+^ T cells, we decided to deliver foreign genes that carry CD8^+^ T cell epitopes into hepatocytes *in vivo* by the HTV administration.

In this study, we have utilized this HTV delivery technology to express a high level of luciferase together with a malaria antigen of interest in the mouse liver. Then, upon a single immunizing dose of rAd expressing a malaria antigen, an immunization regimen known to elicit a robust CD8^+^ T cell response [5], we examined the *in vivo* function of malaria antigen-specific CD8^+^ T cells by the inhibition of the luciferase expression in the liver, as measured by a non-invasive whole body bioluminescent imaging analysis.

We found that a single immunizing dose of AdPyCS and AdPyCelTOS could almost completely inhibit the expression of luciferase in the mouse liver, induced upon HTV delivery of DNAPyCS-Luc and DNAPyCelTOS-Luc, respectively. Furthermore, we were able to determine that the inhibition of luciferase expression by prior immunization with AdPyCS or AdPyCelTOS was mainly due to CD8^+^ T cells, as the depletion of this T cell subset *in vivo* abolished the inhibition of luciferase expression. 

This is an interesting finding in view of our recent observation that although a single immunizing dose of both AdPyCS and AdPyCelTOS could induce a high level of antigen-specific CD8^+^ T cell response, only PyCS-specific CD8^+^ T cell response, but not PyCelTOS-specific CD8^+^ T cell response, was able to inhibit the parasite load in the liver of malaria-challenged mice [15]. Our current results indicate that PyCelTOS-specific CD8^+^ T cells induced by AdPyCelTOS immunization is indeed functional, and that the failure of PyCelTOS-specific CD8^+^ T cells to attack the liver stages of malaria may be due to other factor(s) rather than the function of the CD8^+^ T cells. In order for antigen-specific CD8^+^ T cells to recognize and kill the infected hepatocytes, efficient processing and presentation of malaria antigens onto MHC class I by malaria parasite-infected hepatocytes is necessary. Hence, we speculate that after challenge with sporozoites, PyCelTOS antigen is not sufficiently processed and presented by MHC class I molecules expressed on the infected hepatocytes, thereby preventing CD8^+^ T cells from attacking liver stage malaria parasites. Alternatively, the liver stages of malaria parasites may simply express a lower amount of PyCelTOS. It is important to clarify this issue in the future studies. 

## 5. Conclusions

We believe that our current study demonstrates the successful utilization of a non-viral gene delivery platform that has led to the establishment of a novel method that can assess the *in vivo* function of antigen-specific CD8^+^ T cells in a mouse model. Because of the handling of a non-viral gene, it is a rather simple and safe method, which should be readily applicable for the *in vivo* assessment of the function of antigen-specific CD8^+^ T cells, not only limited to the liver stages of malaria parasites, but also other liver-specific pathogens and cancers. 
